# Glycine protects against high sucrose and high fat-induced non-alcoholic steatohepatitis in rats

**DOI:** 10.18632/oncotarget.12831

**Published:** 2016-10-23

**Authors:** Xin Zhou, Dewu Han, Ruiling Xu, Huiwen Wu, Chongxiao Qu, Feng Wang, Xiangyu Wang, Yuanchang Zhao

**Affiliations:** ^1^ Department of Pathophysiology, Basic Medical Science, Shanxi Medical University, Taiyuan, Shanxi, China; ^2^ Science & technology center of Fenyang College, Shanxi Medical University, Fenyang, Shanxi, China; ^3^ Department of Pathology, Shanxi Provincial People's Hospital, Taiyuan, Shanxi, China; ^4^ Department of Oral Medicine, Shanxi Medical University, Taiyuan, Shanxi, China

**Keywords:** glycine, non-alcoholic steatohepatitis, intestinal endotoxin, endoplasmic reticulum stress, oxidative stress, Pathology Section

## Abstract

We set out to explore the hypothesis that glycine attenuates non-alcoholic steatohepatitis (NASH) in rats and the possible mechanism by which is it does. Male Sprague-Dawley (SD) rats were fed a diet containing high fat and high sucrose (HSHF) for 24 weeks to induce NASH. Blood and liver tissues were sampled at selected time points throughout the study. Compared with control animals, the content of alanine transaminase (ALT), triglycerides (TGs), and free fatty acids (FFAs) in plasma and the TG and FFA content in the liver was increased from week 4 to 24. The level of TNF_α_ and MCP-1 in plasma, the content of TNF_α_ in the liver, the insulin resistance index, inflammatory cell infiltration, hepatocyte apoptosis, reactive oxygen species (ROS) generation, and endoplasmic stress-associated protein expression were unaltered at 4 weeks. However, these levels were significantly elevated in HSHF fed rats at 12 weeks. At the same time, the level of endotoxin progressively increased from 0.08 ± 0.02 endotoxin EU/ml at week 4 to 0.7 ± 0.19 EU/ml at week 24. Moreover, these rats had elevated blood endotoxin levels, which were positively associated with their NASH indexes. Liver histology progressively worsened over the course of the study. However, we found that with concomitant treatment with glycine, the level of endotoxin decreased, while NASH indexes significantly decreased and liver status markedly improved,. These data support the hypothesis that glycine protects against NASH in rats by decreasing the levels of intestinal endotoxin, alleviating endoplasmic reticulum and oxidative stress.

## INTRODUCTION

Nonalcoholic fatty liver disease (NAFLD) is a significant health issue, as it affects up to 30% of adults and up to 10% of children in developed countries [1, 2]. NAFLD is considered one of the manifestations of metabolic syndrome [3]. The NAFLD disease spectrum originates from fatty deposits in the liver; 20% of the deposits progress to develop non-alcoholic steatohepatitis (NASH), which can ultimately result in fibrosis, cirrhosis, liver failure, and even liver cancer [4, 5]. Despite its high prevalence, the factors that influence the transition from fatty liver to NASH remain poorly understood, and no currently available therapies have proven effective [6].

The pathogenesis of NAFLD/NASH has been described by the classic “two-hit theory” [7]. The first hit refers to an accumulation of fatty acids and triglycerides within the liver. The second hit is thought to arise from chronic stresses, such as enhanced lipid peroxidation and increased generation of reactive oxygen species (ROS) [8] and elevated endoplasmic reticulum stress (ERS), the likely byproducts of exacerbated proinflammatory responses in the fatty liver [9]. Clinical observations and experimental studies have found that patients with NAFLD often concomitantly present with intestinal endotoxemia. In support, Harte et al. [10] reported that NAFLD patients frequently have increased circulating endotoxin levels. Studies conducted by Brun et al. [11] have shown that elevated intestinal permeability can lead to a greater incidence of endotoxemia in genetically obese mice, which can cause inflammatory liver damage. Recent studies have demonstrated that endotoxin is a potential source of ROS and endoplasmic reticulum stress (ERS) [12, 13]. These studies imply that inhibition of endotoxin may be an effective treatment for NAFLD/NASH. Studies have demonstrated that the amino acid glycine has anti-inflammatory, cell protective, and immunomodulatory properties. Glycine can attenuate liver injury by inhibiting the activity of kupffer cells and the production TNFα [14], as well as down-regulating TLR4 signaling [15]. Based on these results, we hypothesized that glycine may protect against liver injury by reducing endotoxin, and we set out to determine if glycine can inhibit oxidative stress and endoplasmic reticulum stress.

We have successfully established a reproducible animal model of NAFLD induced by a high fat and high sucrose (HSHF) diet along with intestinal endotoxemia [16]. Therefore, the goal of our current work was to determine if glycine can protect against liver injury by decreasing the level of endotoxemia and attenuating oxidative and endoplasmic reticulum stress.

## RESULTS

### Changes of biochemistry, inflammation, and pathology in a NASH rat model

Significant differences in the ALT, TG, and FFA levels in plasma, as well as liver homogenates, were noted by week 4 and reached their height by week 12; these differences continued to 24 weeks (Table 1). At 24 weeks, plasma levels of TGs (*p* < 0.001) and FFAs (*p* < 0.001) and liver homogenate levels of TGs (*p* < 0.001) and FFAs (*p* < 0.001) were higher in the H (high sucrose, high fat diet) group compared to the C (control) group (Table 1).

As shown in Table 1, serum lipopolysaccharide (LPS), TNFα, and MCP-1 levels and liver TNFα levels and HOMA-IR increased significantly by the 12^th^ week in H rats compared to C rats, and this difference persisted up to 24 weeks. This suggests that intestinal endotoxemia occurred by the 12^th^ week and continued as long as 24 weeks. At the same time, chronic inflammation and insulin resistance became apparent in H rats. Correlation analysis showed that the level of LPS in plasma was progressively elevated in NASH rats, which was positively related to elevated HOMA-IR, elevated levels of ALT, TNF-α, MCP-1 in plasma, and elevated levels of TGs, FFAs, and TNF-α in liver homogenates (Figure 2A-2C).

**Table 1:** Changes in biochemistry and inflammation in the NASH rat model

	Control	4w	12w	24w
LPS(EU/ml)	0.07±0.02	0.08±0.02	0.61±0.07^a^^b^	0.70±0.19^a^^b^
ALT(U/L)	25.88±2.25	69.86±9.43^a^	259.79±26.11^a^^b^	308.66±24.38^a^^b^
TG (mmol/L)	0.08±0.01	0.23±0.01^a^	0.61±0.13^a^^b^	0.55±0.07^a^^b^
FFA(mmol/L)	395.9±22.36	471.67±38.41^a^	899.48±130.11^a^^b^	1266.33±243.61^a^^b^^c^
TG in liver(mmol/g)	0.91±0.21	1.13±0.07^a^	1.39±0.28^a^^b^	1.41±0.14^a^^b^
FFA in liver(umol/L)	82.81±3.47	121.27±18.77^a^	225.81±36.18^a^^b^	248.32±28.06^a^^b^
HOMA-IR	4.10±0.73	4.60±0.76	23.82±8.89^a^^b^	44.47±13.68^a^^b^^c^
TNFα(ng /ml)	1.10±0.09	1.18±0.07	2.01±0.21^a^^b^	2.05±0.41^a^^b^
TNFα in liver(ug / L)	0.08±0.007	0.09±0.004	1.39±0.28^a^^b^	1.41±0.14^a^^b^
MCP-1(ng/L)	4.90±0.58	5.32±0.63	19.33±0.9^a^^b^	20.10±0.93^a^^b^

Standard control group (C), 4th week group (4w), 12th week group (12w), 24th week group (24w).

a *P* < 0.05 *vs* standard control group;

b *P* < 0.05 *vs* 4^th^ week HSHF group;

c *P* < 0.05 *vs* 12^th^ week HSHF group.

### Changes in pathology, apoptosis, staining for CD68^+^, ROS, and the expression of p-JNK1 /JNK1, IKKβ, GRP78, and CHOP in the livers of NASH rats

Haematoxylin and eosin (H&E) staining showed gradual increases in fat degeneration, ballooning degeneration, and lobular and periportal inflammatory cell infiltration in H rats compared to C rats from the 4^th^ to 24^th^ week; fibrosis was gradually increased by the 24^th^ week as well (Figure 1.1A-1.1D).

Terminal deoxynucleotidyl transferase dUTP nick end labeling (TUNEL) of normal liver tissue showed very little hepatocyte apoptosis. However, hepatocyte apoptosis significantly increased at 12 weeks and continued to increase until 24 weeks in the H group (Figure 1.2A-1.2E). Normal liver tissue showed a very small number of infiltrating CD68-positive macrophages. However, liver tissue infiltration increased by the 12^th^ week and peaked by the 24^th^ week in the H group (Figure 1.3A-1.3E). ROS expression in the liver gradually increased from the 4^th^ to the 24^th^ week as displayed in Figure 1.4A-1.4E.

**Figure 1:**
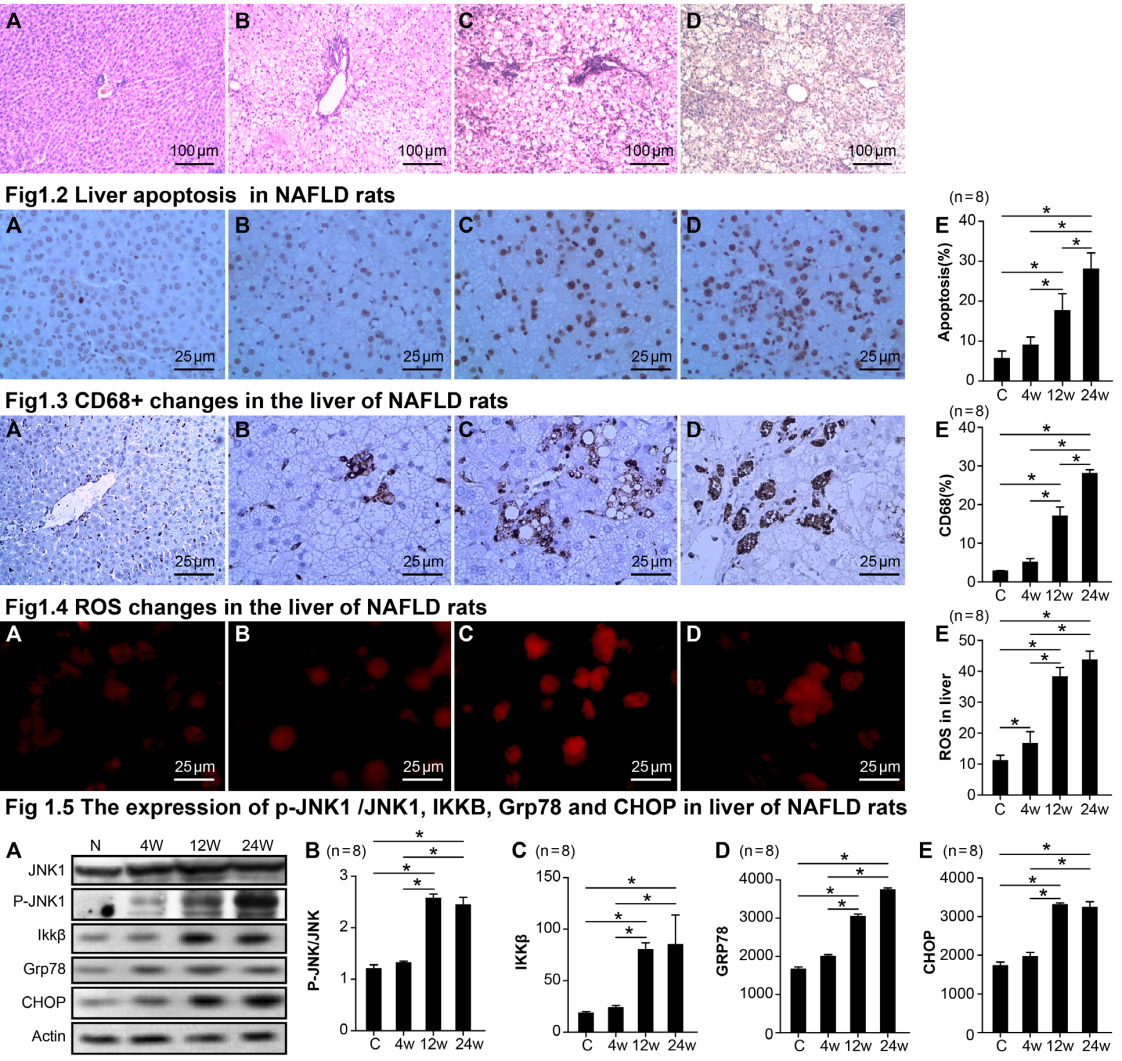
Changes in pathology, apoptosis, CD68^+^, ROS, the expression of p-JNK1 /JNK1, IKKβ, GRP78, and CHOP in NASH rats Fig1.1 Pathological changes in the liver of NASH rats. control group **A.**, 4th week group **B.**, 12th week group **C.**, 24th week group **D.**. Fig1.2 Liver apoptosis in NASH rats. control group **A.**, 4th week group **B.**, 12th week group **C.**, 24th week group **D.**, statistical analysis of apoptosis **E.**. Fig1.3 CD68^+^ changes in the liver of NASH rats. control group **A.**, 4th week group **B.**, 12th week group **C.**, 24th week group **D.**, statistical analysis of CD68^+^ staining. **E.**. Fig1.4 ROS changes in the liver of NASH rats. control group **A.**, 4th week group **B.**, 12th week group **C.**, 24th week group **D.**, statistical analysis of CD68^+^ staining. **E.**. Fig 1.5 The expression of p-JNK1 /JNK1, IKKB, Grp78, and CHOP in the livers of NASH rats. The protein expression of JNK1, p-JNK, IKKB, Grp78, and CHOP in livers *via* Western Blot **A.**, statistical analysis of p-JNK/ JNK1expression levels in NAFLD rats **B.**, statistical analysis of IKKB expression levels in NAFLD rats **C.**, statistical analysis of Grp78 expression levels in NAFLD rats **D.**, statistical analysis of CHOP expression levels in NAFLD rats **E.**. Data represents means ± standard error (*n* = 8). “*”indicates a statistically significant difference (*P* < 0.05).

**Figure 2:**
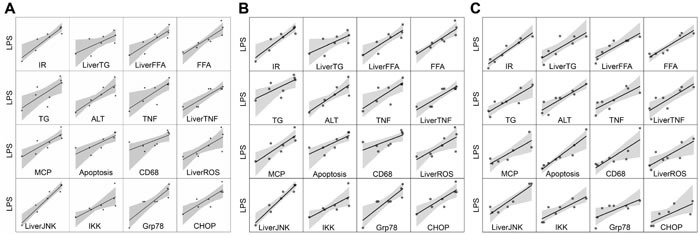
Correlation analysis of LPS *vs* IR, TG, FFA, ALT, TNFα, MCP-1, Apoptosis, CD68, ROS, P-JNK/JNK, IKK β, GRP78, and CHOP Correlation analysis of LPS *vs* indexes of NASH rats in 4 weeks **A.**. Correlation analysis of LPS *vs* indexes of NASH rats in 12 weeks **B.**. Correlation analysis of LPS *vs* indexes of NASH rats in 24 weeks **C.**.

We found that a high fat and high sucrose diet led to a significant increase in the expression ratio of p-JNK1/JNK1 in the liver by 12 weeks (an index of JNK1 activation; *p* < 0.001; Figure 1.5A-1.5E), yet there was no change in total JNK. Similarly, IKKβ, GRP78, and CHOP were also elevated in the H group by 12 weeks (Figure 1.5A-1.5E). Correlation analysis showed that the level of LPS in the plasma of NASH rats was also positively related to the up-regulation of apoptosis, CD68, ROS, P-JNK1/JNK1, IKKβ, GRP78, and CHOP in liver homogenates (Figure 2A-2C).

### Effect of glycine treatment on biochemistry and systemic inflammation

**Figure 3:**
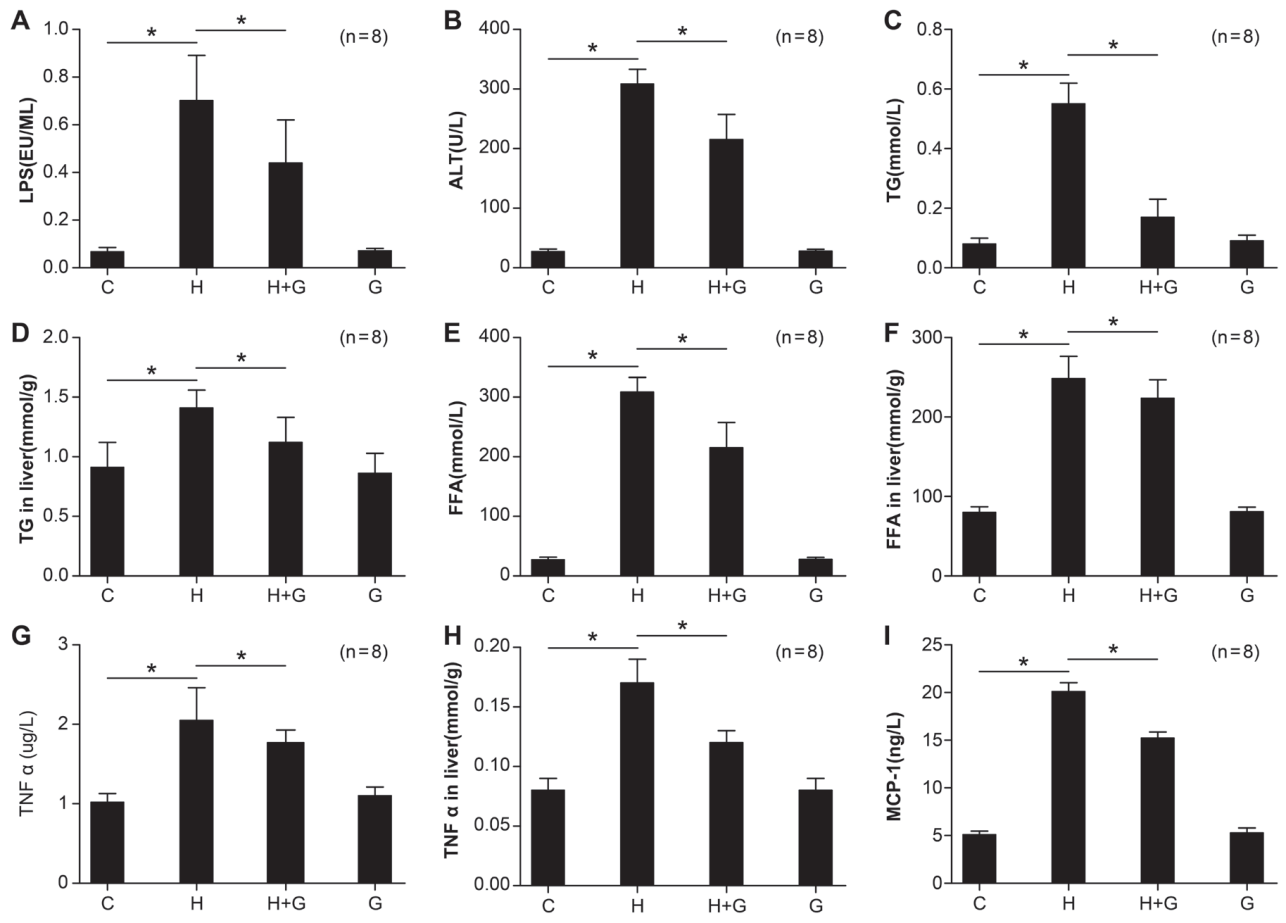
Effect of glycine treatment on LPS, biochemistry, and systemic inflammation indexes Standard control group (C), high fat and high sugar group (H), high fat and high sugar + glycine group (H+G), glycine group (G). Data represents mean ± standard error (*n* = 8). “*”indicates a statistically significant difference (*P* < 0.05).

To investigate the protective role of glycine in high fat, high sucrose-induced NASH, rats were treated with glycine concomitantly to feeding with sucrose or water. Portal endotoxin was significantly reduced in the H group treated with glycine (H+G group) compared to rats only fed the HSHF diet (Figure 3). At the same time, with the treatment of glycine, the level of inflammatory factors, such as TNFα and MCP-1 in serum and TNFα in the liver significantly decreased in the H+G group (Figure 3). The content of ALT, TGs, and FFAs in serum and TGs and FFAs in the liver was significantly reduced in H+G rats compared to those fed an HSHF diet alone (Figure 3). These data suggest that glycine treatment might attenuate intestinal endotoxemia, improve liver function, alleviate hyperlipidemia, and at least partially relieve the inflammatory response.

### Effect of glycine treatment on liver pathology

**Figure 4:**
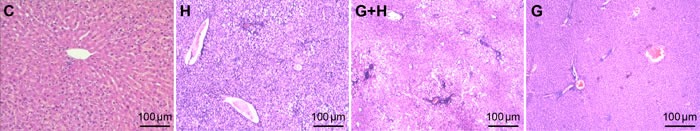
Effect of glycine treatment on liver pathology Standard control group (C), high fat and high sugar group (H), high fat and high sugar + glycine group (H+G), glycine group (G).

To investigate liver pathology, we used H&E staining and examined liver pathology changes under a light microscope. There were no significant changes in the livers of normal rats *versus* glycine rats (Figure 4). However, in the HFHS group, morphologic changes were present reflective of fatty and ballooning degeneration, and the lobular and periportal structures exhibited inflammatory cell infiltration (Figure 4). However, liver injury was obviously decreased in the livers of mice receiving glycine treatment (Figure 4).

### Effect of glycine treatment on hepatocyte apoptosis

**Figure 5:**
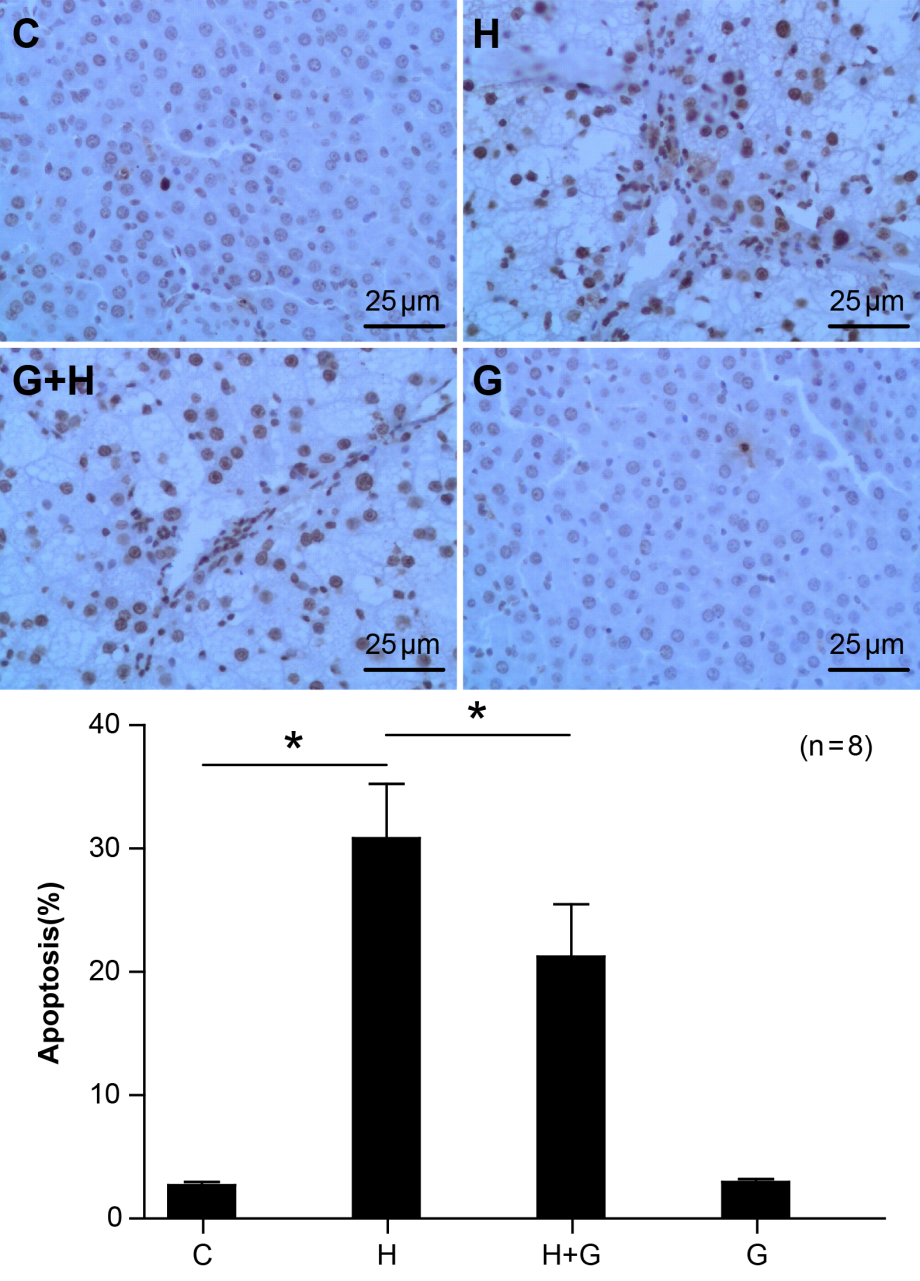
Effect of glycine treatment on hepatocyte apoptosis Standard control group (C), high fat and high sugar group (H), high fat and high sugar + glycine group (H+G), glycine group (G). “*”indicates a statistically significant difference (*P* < 0.05). Data represents mean ± standard error (*n* = 8).

As hepatocyte apoptosis is a signature of nonalcoholic fatty liver disease, we investigated apoptosis is liver samples using TUNEL. There was minimal hepatocyte apoptosis in the C group (Figure 5). However, a high sugar and high fat diet significantly elevated hepatocyte apoptosis in the NASH group (Figure 5). However, upon treatment with glycine, there were marked decreases in the number of TUNEL-positive staining hepatocytes when compared to the HFHS group (Figure 5). These data suggest that glycine treatment might protect against NASH by attenuating liver apoptosis.

### Effect of glycine treatment on macrophage infiltration

**Figure 6:**
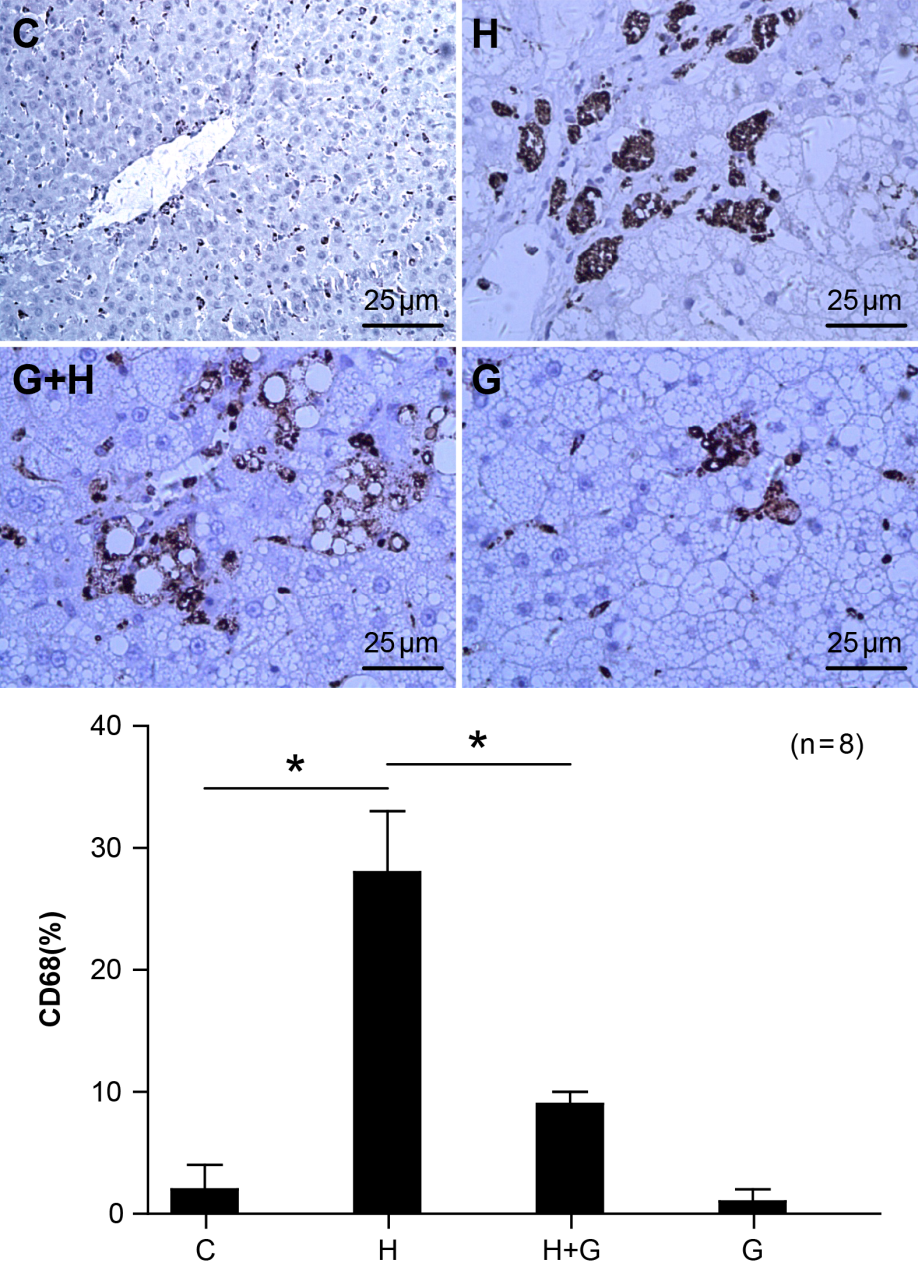
Effect of glycine treatment on macrophage infiltration Standard control group(C), high fat and high sugar group (H), high fat and high sugar + glycine group (H+G), glycine group (G). “*”indicates a statistically significant difference (*P* < 0.05).Data represents mean ± standard error (*n* = 8).

CD68 is a macrophage marker. We detected CD68 protein in liver tissue by immunohistochemistry. The CD68 staining assay showed that expression of CD68 was up-regulated by a high fat and high sugar diet (Figure 6). However, the expression of CD68 was significantly down-regulated after treatment with glycine in NASH rats (Figure 6). There were no significant differences between the control and glycine group. These results suggest that glycine may attenuate macrophage infiltration and the inflammatory response.

### Effect of glycine treatment on oxidative stress

**Figure 7:**
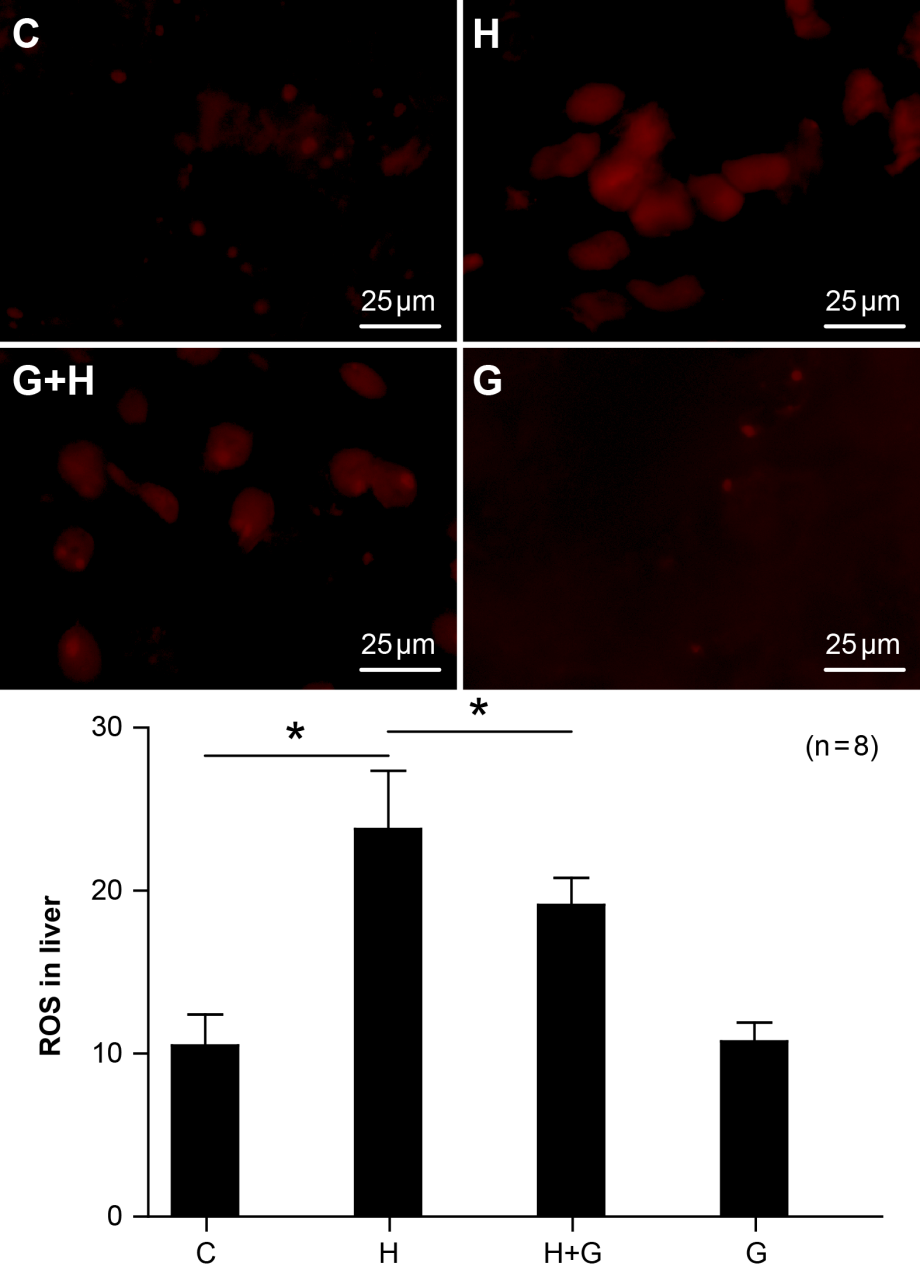
Effect of glycine treatment on oxidative stress Standard control group (C), high fat and high sugar group (H), high fat and high sugar + glycine group (H+G), glycine group (G). “*”indicates a statistically significant difference (*P* < 0.05). Data represents mean ± standard error (*n* = 8).

Oxidative stress is one factor involved in the the pathogenesis of NASH. Therefore, we looked for ROS changes in the liver. In the HSHF group, the expression of ROS was markedly increased (Figure 7). However, ROS expression was significantly down-regulated with glycine treatment (Figure 7). There were no significant differences between the control and glycine group (Figure 7). Therefore, our results demonstrate that glycine treatment can decrease oxidative stress induced by a high fat and high sugar diet in NASH rats.

### Effect of glycine treatment on endoplasmic reticulum stress

**Figure 8:**
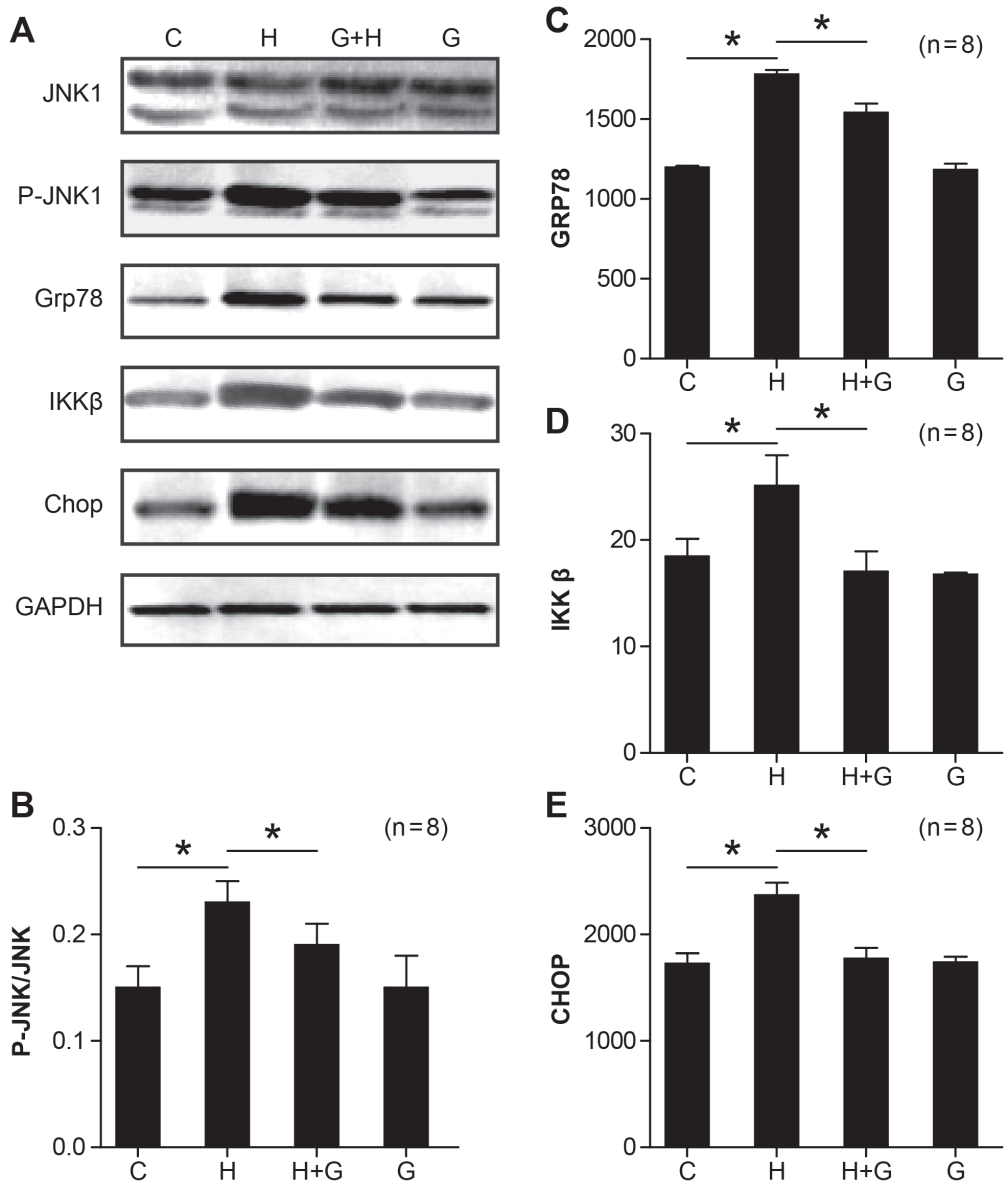
Effect of glycine treatment on endoplasmic reticulum stress Standard control group (C), high fat and high sugar group (H), high fat and high sugar + glycine group (H+G), glycine group (G). “*”indicates a statistically significant difference (*P* < 0.05). Data represents mean ± standard error (*n*= 8).

Endoplasmic reticulum stress (ERS) is another factor involved in the pathogenesis of NASH. Therefore, we examined the expression of ER stress markers. As shown in Figure 8, the expression of ER stress-related molecules, such as JNK1, Grp78, IKKβ, and Chop were increased in NASH rats (Figure 8). However, the protein expression of p-JNK1, Grp78, IKKβ, and Chop were significantly reduced by glycine treatment (Figure 8). There were no obvious changes between the control and glycine group (Figure 8). These results suggest that glycine can attenuate the ER stress in NASH rats.

## DISCUSSION

It has been reported that the development of NASH is accompanied by increased LPS plasma levels [10, 17], which possibly occur through mechanisms involving increased intestinal permeability, small intestinal bacterial overgrowth (SIBO), tight junction alteration, and bacterial translocation [18-23]. We previously set up an animal model of metabolic syndrome with NAFLD and type two diabetes mellitus (T2DM), in which intestinal endotoxemia occurs spontaneously [16]. Increasing numbers of studies have demonstrated that endotoxin is a potential inducer of ROS and ERS [24, 25]. In the liver, LPS can bind receptors (e.g.CD14/TLR-4) that activate the NF-КB pathway by de-repressing/activating IKKβ, which in turn increases NADPH oxidase activity by stimulating Kuppfer cell infiltration in the liver [26]; this results in the release of large amounts of ROS. Concomitantly, LPS levels may increase the level of circulating free fatty acids (FFAs) *via* the lipolysis of adipocytes [27]. When FFA levels are severely elevated in the liver, FFAs may be converted to triglycerides and accumulated in liver cells [28], which may significantly enhance the catalyticase activity of the lipid peroxidases CYP2E1 and CYP4A. The induction of the latter plays an important role in both FFA microsome ω-oxidation and peroxisome β-oxidation, which may produce an excessive amount of ROS metabolites [24].

The endoplasmic reticulum (ER) is an important organelle, which is a reservoir for calcium ions in eukaryotic cells; it is also a key location where protein synthesis, processing, and folding occur [29]. It has been confirmed that endotoxin is an important ER stressor [25]. In addition, ER stress may be indirectly induced by accumulating oxidative stress-associated molecules [30]. Now, ER stress may be considered as the inflammatory basis of metabolic disease [31]. ER stress can activate a variety of inflammatory pathways, such as the JNK and IKKβ pathway. The interaction of these pathways induces expression of inflammatory cytokines. IR, which is a byproduct of ER stress, is mainly produced *via* activation of IRE1α-mediated JNK [32]. Furthermore, endoplasmic reticulum stress can lead to liver cell apoptosis through JNK, caspase, and the CHOP apoptosis pathway. These results show that oxidative stress and endoplasmic reticulum stress are the basis of the inflammation, insulin resistance, and apoptosis that occurs during the development of NASH [33-35]. Correlation analysis showed that increased endotoxin levels were positively correlated to the enhanced content of ALT, TG, FFA, TNF-α, MCP-1, and HOMA-IR in plasma and TG, FFA, TNF-α, ROS, apoptosis, CD68, p-JNK1/JNK1, IKKβ, GRP78, and CHOP in the liver. These findings suggest that increases in LPS levels are correlated with the changes that occur in NASH rats, such as inflammatory cell recruitment, hepatocyte apoptosis, oxidative stress, and endoplasmic reticulum stress.

Glycine is one of the nonessential amino acids and is the simplest amino acid. Many research studies have reported that glycine has antagonistic effects on endotoxin. Yang et al. found that elevated levels of TNFα in plasma can be effectively inhibited by intravenous injection of glycine during the early stages of sepsis [36]. Ikejima et al. showed that 5% glycine in the diet of rats can inhibit elevated TNFα and ALT and decrease hepatic necrosis in rat models of endotoxin shock [37]. In a complex factor-induced liver fibrosis rat model, Liu et al. found that glycine can reduce endotoxin levels and delay the occurrence and development of cirrhosis [38]. Han et al. confirmed that glycine can reduce endotoxin and liver damage through the following mechanisms: 1) glycine can reduce intestinal permeability and reduce the absorption of endotoxin, 2) glycine is capable of binding the lipid A portion of endotoxin and reducing endotoxin activity, and 3) glycine can decrease the expression of lipopolysaccharide binding protein (LBP) and CD14 mRNA, thereby reducing the excessive activation of KC (murine homology of IL-8). In addition, glycine has been reported to inhibit hepatocyte apoptosis [39] and reduce oxidative stress [40].

In the present study, our experiment confirmed that glycine can reduce plasma endotoxin levels, while ROS content, endoplasmic reticulum stress-related protein levels, and liver lesions improved after glycine treatment. Therefore, we believe that intestinal endotoxemia is involved in the pathogenesis of diet-induced NAFLD, and glycine protects against liver injury by reducing the levels of endotoxin to inhibit oxidative stress and attenuate endoplasmic reticulum stress.

However, there are several questions remainingMoreover,

**Figure 9:**
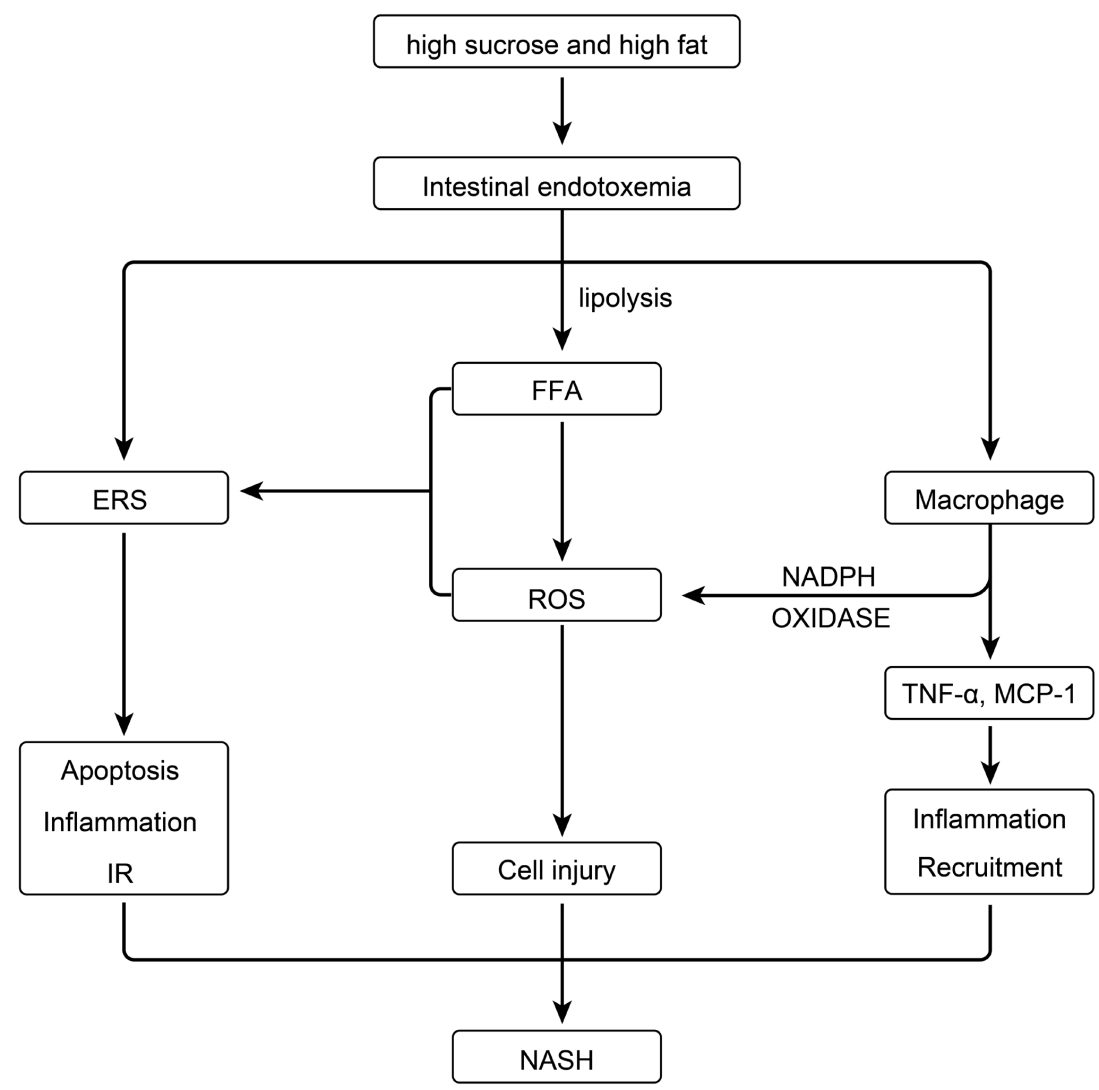
Effect of intestinal endotoxemia in the pathogenesis of NASH

## MATERIALS AND METHODS

### Animals and treatments

Fourty-eight male Sprague-Dawley rats weighing 200-250 g were obtained from the Animal Center of Shanxi Medical University. The experimental animals were randomly divided into four groups (C, H, H+G, and G group). The C group (control group, *n* = 8) received regular diet and tap water; the H group (high sucrose and high fat group, *n* = 24) received high sucrose and high fat diet (HSHF; 52% of calories from carbohydrates, 25% from fat, and 10% from protein); the H+G group (high sucrose and high fat + glycine, *n* = 8) received the HSHF diet and 3.5 g/kg of glycine per day, while the G group (glycine, *n* = 8) received 3.5 g/kg of glycine per day and a regular diet [41]. The H group was divided into the following groups (*n* = 8 per group): 4, 12, and 24 weeks. Animals and the rodent diet (standard rodent diet) were obtained from the Research Animal Center of Shanxi Medical University. The animals were housed under standard laboratory conditions, maintained on a 12 h light and dark cycle and had unrestricted access to food and water. The experimental protocols were approved by the Shanxi Animal Research Ethics committee.

Rats were sacrificed at 4, 12, and 24 weeks accordingly. The blood and liver tissues of rats were sampled at the time of sacrifice (4, 12, or 24 weeks) and were stored at -80°C until processing.

### Measurements of serum endotoxin levels in the abdominal aorta

In anesthetized animals, sterile operation and blood samples were collected from the abdominal aorta and centrifuged at 1000g for 10 min. The levels of endotoxin in the collected plasma were determined using a Limulus amebocyte lysate regent kit (Clinical Sciences Inc, Xiamen China) according to the manufacturer's instructions (UV-2102C, Shanghai).

### Measurement of TNF-α, MCP-1, alanine transferase, TGs, FFAs in plasma, and TNF-α, TG, and FFAs in liver homogenates

The amount of TNF-α (Tumor necrosis Factor-α, TNF-α radioimmunoassay kit, Radioimmunity Institute of PLA General Hospital, Beijing China), MCP-1(Monocyte chemoattract NT Protein-1, MCP-1 ELISA Kit, Hysen Bear Technology limited company,Shanghai China), ALT (alanine transferase kit, Nanjing Jiancheng Bioengineering Institue,Nanjing China), TGs (Triglyceride kit, Nanjing Jiancheng Bioengineering Institue,Nanjing China) and FFAs (free fatty acid kit, Nanjing Jiancheng Bioengineering Institue,Nanjing China) in the plasma and the amount of TNF-α, TGs, and FFAs in the liver were measured following the manufacturer's instructions.

### Radioimmunoassay for insulin and HOMA-IR

Plasma insulin concentrations were determined using an insulin RIA kit (Poor Albert Biotechnology Technology Coporation, Beijing China). The HOMA-IR (homeostasis model assessment of insulin resistance) index reflects the degree of insulin resistance and was calculated as fasting serum glucose times fasting serum insulin over a factor of 22.5 [42].

### Liver histology

Liver samples were fixed in 10% formalin, embedded in paraffin wax, cut into 4 μm thick sections, and stained with hematoxylin and eosin (HE, Hematoxylin and eosin staining kit, Junruishengwu Technnology Corporation, Shanghai China). Frozen sections of liver were sliced and stained with Sudan IV(Sudan IV fat staining kit, Junruishengwu Technnology). To estimate the relative inflammation, steatosis, and fibrosis of the liver, hepatic sections were studied under light microscopy (Olympus BX51 microscope).

### Immunohistochemistry

Paraffin-embedded sections were stained with CD68 protein adducts using a polyclonal antibody (1:100 dilution; Zhong Shan-Golden Bridge Biological Technology Co. Beijing, China) to observe quantitative expression levels of CD68.

The specimens were analyzed using a computerized image analysis system (IPP6.0 software, Media Cybernetics Inc., USA). Two independent observers were blinded to treatment groups when analyzing CD68 staining. The cells where the cytoplasm stained brown, as observed under an optical microscope, were defined as positive cells. The hepatocytes and macrophages were calculated from 8-10 fields, and the percent of positive cells was calculated as followed: number of CD68 positive cells / number of total hepatocytes counted. (at 400X)

### Terminal deoxynucleotidyl transferase-mediated dUTP nicked end labeling (TUNEL) assay

For *in situ* detection of apoptosis in the liver, parraffin wax sections were dewaxed to water and washed in 0.01 M PBS (pH 7) twice for 5 min, flooded with 3% hydrogen peroxide for 20 minutes, and finally washed with PBS twice for 5 min and dried. Fifty μl of reaction solution (TUNEL kits, Roche Diagnostics GmbH, Germany) was added to each sample, which was then incubated at 37°C for 60 min in the dark under humid conditions. The samples were then washed twice for 5 min in PBS. Fifty μl Converter-POD (TUNEL kits, Roche Diagnostics GmbH, Germany) was added to each sample, which was incubated at 37°C for 30 min and washed twice in PBS and stained with DAB Horseradish Peroxidase (DAB Horseradish Peroxidase Color Development Kit, Beyotime Institute of Biotechnology, Shanghai China). Finally, the samples were hematoxylin stained and underwent conventional dehydration and were transparently mounted with glycerol. In the subsequent images, nuclear staining was considered positive. The specimens were analyzed using a computerized image analysis system (IPP6.0 software, Media Cybernetics Inc, USA), and the percent of positive cells was calculated as followed: number of TUNEL positive cells / number of total hepatocytes counted. (at 400X)

### ROS levels in liver

Frozen livers were allowed to obtain an equilibrium temperature of -20°C and then frozen blocks were OTC embedded. Five μm serial sections were prepared. Sections were stained with dihydroethidium (DHE; 1:1000 dilution, Sigma-Aldrich, St. Louis, MO), incubated for 15 min at room temperature in the dark, and washed 3 times with phosphate-buffered saline (PBS). Sections were mounted in 50% glycerol, and ROS levels in the liver were observed under a fluorescence microscope. To determine means, data were recorded as the number of ROS-positive cells per field (at 400X) in each tissue section.

### Western blot analysis

Total JNK1, p-JNK1, IKKβ, Grp78, and CHOP were assessed by Western blot. Aliquots of frozen liver homogenates were further extracted in phosphate-buffered saline containing of 1% NP-40, 0.5% sodium deoxycholate, 0.1% SDS, 0.1 mM EDTA, 50 mM NaF, and 2 mM Na_3_VPO_4_ (NaF and Na_3_VPO_4_ only for phosphorylation protein) and lysed by 30 min incubation on ice. Chemicals came from Nanjingjiancheng Biotechnology, Nanjing China. The lysate was centrifuged at 15,000 rpm for 10 min. Forty μg of protein was loaded in each lane and separated on a 7.5% sodium dodecyl sulfate polyacrylamide gel (SDS-PAGE) and then transferred to a polyvinylidene difluoride (PVDF) membrane. Blots were blocked for 3 h at room temperature with 5% (w/v) non-fat dried milk. After washing 3 times with TBST (Tris 50mmol/L, NaCl 100 M mmol/L, pH 7.4), the membrane was incubated at 4°C overnight with specific antibodies. Rabbit polyclonal antibodies against JNK1 (1:1000 dilution; Cell Signaling Technology Inc. Danvers, USA), p-JNK1 (1:750 dilution; Cell Signaling Technology Inc. Danvers, USA), IKKβ (1:1000 dilution, Cell Signaling Technology), Grp78 (1:1000 dilution; Cell Signaling Technology), and CHOP (1:500 dilution; Santa Cruz Biothechnology Inc. CA, USA) were used in the study. The immunoblots were then incubated with the corresponding peroxidase-conjugated goat anti-rabbit secondary antibodies (1:2000 dilution; Zhong Shan-Golden Bridge Biological Technology). The bands were detected with enhanced chemiluminescence. The intensities of the protein bands were analyzed by Quantity One software (Bio-Rad Laboratories, Inc. Hercules, USA).

### Statistics

All values are displayed as mean ± standard error, and statistical analyses were performed by SPSS13.0 system (Statistical Product and Service Solutions, USA). The Spearman correlation coefficient was calculated to assess the relationship between the levels of plasma endotoxin and ALT, TG, FFA, TNF_α_, MCP-1, HOMA-IR, ROS, apoptosis, CD68, p-JNK/JNK, IKKβ, Grp78, and CHOP. Other data were analyzed by one-way analysis of variance (ANOVA). The statistical significance level was set at *P* < 0.05.
